# Camptocormia in patients with multiple system atrophy at different disease durations: frequency and related factors

**DOI:** 10.1186/s12883-021-02210-y

**Published:** 2021-04-28

**Authors:** Ling Yu Zhang, Bei Cao, Qian-Qian Wei, Ru Wei Ou, Bi Zhao, Jing Yang, Ying Wu, Hui Fang Shang

**Affiliations:** Department of Neurology, Laboratory of Neurodegenerative Disorders, Rare Diseases Center, West China Hospital, Sichuan University, Chengdu, 610041 Sichuan China

**Keywords:** Multiple system atrophy, Camptocormia, Disability, Cohort study

## Abstract

**Background:**

Camptocormia is common in patients with multiple system atrophy (MSA). The current study was aimed at assessing the frequency of camptocormia and its related factors in MSA patients with different disease durations. Also, the impact of camptocormia on disability was evaluated.

**Methods:**

A total of 716 patients were enrolled in the study. They were classified into three groups based on disease duration (≤ 3, 3–5, ≥ 5 years). Specific scales were used to evaluate the motor and non-motor symptoms. Disease severity was assessed using the Unified Multiple System Atrophy Rating Scale (UMSARS). The binary logistic regression model was used to explore the factors related to camptocormia. To analyze the impact of camptocormia on disability in patients with disease duration less than 5 years, propensity score matching (PSM) and stratified Cox regression analysis were used.

**Results:**

In the current study, we found that the frequency of camptocormia was 8.9, 19.7 and 19.2% when the disease duration was ≤3, 3–5, ≥ 5 years, respectively. In the disease duration ≤3 years group, we found that MSA-parkinsonian subtype (MSA-P) (OR = 2.043, *P* = 0.043), higher total UMSARS score (OR = 1.063, *P* < 0.001), older age of onset (OR = 1.047, *P* = 0.042), and lower score on the frontal assessment battery (FAB) (OR = 0.899, *P* = 0.046) were associated with camptocormia. Only greater disease severity was associated with camptocormia in the group of patients with disease duration 3–5 years (OR = 1.494, *P* = 0.025) and in the group of patients with disease duration ≥5 years (OR = 1.076, *P* = 0.005). There was no significant impact of camptocormia on disability in patients with a disease duration of < 5 years (HR = 0.687, *P* = 0.463).

**Conclusion:**

The frequency of camptocormia increased with prolonged disease duration. Disease severity was related to camptocormia at different stages of the disease. The MSA-P subtype, older age of onset, and lower FAB score were associated with camptocormia in the early stage of the disease.

## Introduction

Camptocormia is an axial postural deformity characterized by abnormal thoracolumbar spinal flexion that occurs while standing or walking and abates or disappears in the supine position [1]. Skidmore et al. described a patient with pathologically proven multiple system atrophy (MSA) who presented with parkinsonism and camptocormia [2]. It has been reported that camptocormia is a common but under-recognized problem in patients with MSA besides Parkinson’s disease (PD). This causes marked functional disability which is independent of other motor symptoms [3]. Furthermore, camptocormia was proposed as a supporting feature (red flag) in the diagnosis of MSA [4]. However, the pathogenesis of camptocormia is not well understood and might be related to axial dystonia, myopathy, and disproportionate rigidity [5].

In addition, considerably less attention has been accorded to the frequency or related factors of camptocormia in MSA. The frequency of camptocormia of MSA has been reported in several studies [3, 6, 7] exception of some case reports [2, 8, 9]. Ashour and Jankovic found camptocormia in 26.3% of the 19 patients with MSA. Koellensperger et al. reported that the frequency of camptocormia was 32.1% in 74 patients with MSA-P [4]. Recently, camptocormia and/or Pisa syndrome was found in 5 (3.1%) of the 160 patients with pathologically confirmed MSA [7]. Earlier, we had found that the frequency of camptocormia was 15.6% in Chinese patients with MSA [10]. The varied frequency of camptocormia that has been reported across separate studies might be attributed to the difference in sample size and the heterogeneity in the enrolled patients with MSA.

However, the factors associated with camptocormia in patients with MSA remain largely unknown. Our previous study found that the disease severity was associated with camptocormia in MSA [10]. However, it is unclear whether non-motor symptoms, such as cognition, depression, and anxiety, are associated with camptocormia. Additionally, the frequency of camptocormia and related factors associated with camptocormia among different disease stages remain unknown. Furthermore, it has been reported that MSA patients have a poor prognosis with the median time from the onset to the requirement of an aid for walking, wheelchair requirement, and a bedridden state being three, five, and 8 years, respectively [11]. The relationship between camptocormia and disability is unknown. Therefore, in the present study, we aimed to explore the frequency of camptocormia, the potential factors related to camptocormia, and whether camptocormia is a predictor of disability in a large cohort of MSA patients with different disease durations wherein the motor and non-motor symptoms were comprehensively assessed by specific scales.

### Patients and methods

We registered patients who were consecutively recruited from the Department of Neurology at the West China Hospital of Sichuan University between January 2014 and December 2019. Patients who met the “probable” diagnosis of MSA [12] were enrolled in the current study. A total of 209 patients who met the “possible” diagnosis of MSA (158/209) or had incomplete data (51/209) were excluded from the current study. Patients were screened for spinocerebellar ataxia (SCA) genes, including SCA1, 2, 3, 6, and 7, to exclude the common forms of SCA. They were also subjected to brain MRI scans to exclude other neurological disorders.

**Fig. 1:**
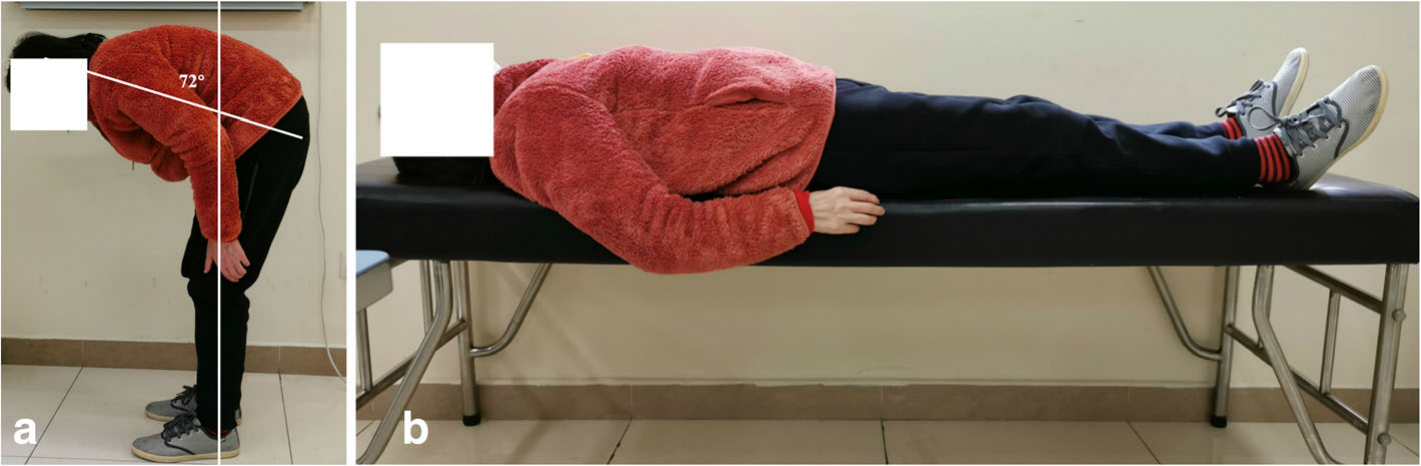
MSA patient with camptocormia when standing (**a**) and completely alleviated in the supine position (**b**)

All patients were evaluated during face-to-face interviews with neurologists. Clinical information regarding sex, age, age of onset, symptom of onset, disease duration, levodopa equivalent daily dose (LEDD), and years of education were collected. The disease duration was defined as the time from the symptom of onset to the evaluation date. MSA patients with predominantly parkinsonian features were designated as MSA-P, and patients with predominantly cerebellar ataxia were designated as MSA-C [12]. Symptom of onset was defined as the initial presentation of any motor symptoms (i.e., parkinsonism or cerebellar ataxia) or autonomic features, except for male erectile dysfunction [12]. Camptocormia was defined as at least 45° thoracolumbar flexion that was apparent when standing or walking; which abated or disappeared in the supine position [1]. The degree of thoracolumbar flexion was assessed by calculating the angle between the vertical plane and a line passing through the trochanter and the edge of the acromion [13] (Fig. 1). Disability was used to refer to the patients who were always confined to a wheelchair. The disability time was defined as the interval from the date of symptom onset to the date of confinement to a wheelchair.

The disease severity was assessed using the Unified Multiple System Atrophy Rating Scale (UMSARS) [14]. Orthostatic hypotension (OH) was defined as a drop in systolic blood pressure ≥ 30 mmHg and/or diastolic blood pressure ≥ 15 mmHg. The frontal assessment battery (FAB) was used to assess the frontal lobe function [15]. The Montreal Cognitive Assessment (MoCA) was used to assess global cognition [16]. The severity of depression was evaluated using the Hamilton Depression Rating Scale-24 (HDRS-24) [17]. The severity of anxiety was assessed using the Hamilton Anxiety Rating Scale (HARS) [18].

**Fig. 2:**
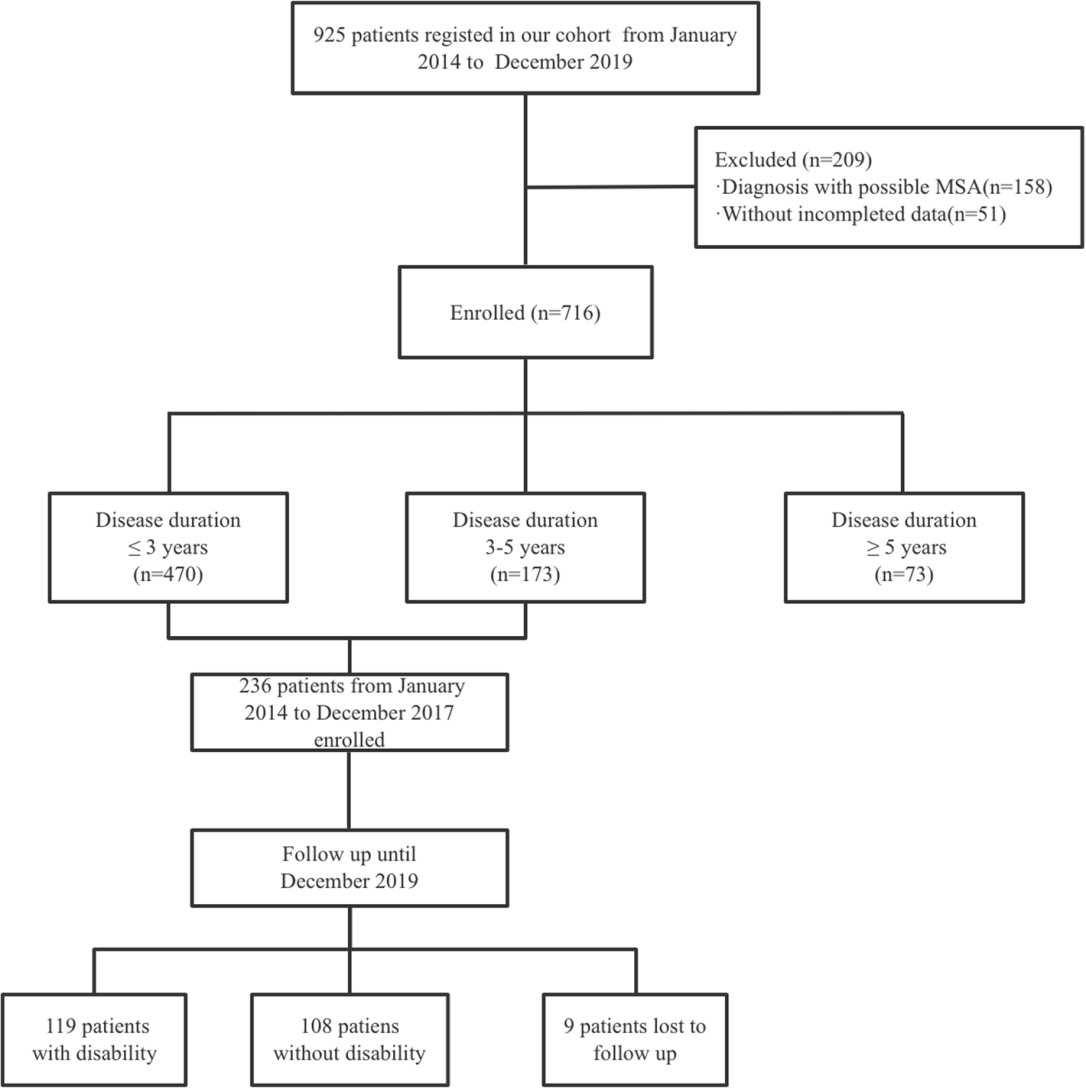
Study flow diagram

A total of 716 patients with MSA were enrolled in the current study. MSA patients were divided into three groups: disease duration ≤3 years group, disease duration 3–5 years group, and disease duration ≥5 years group according to disease duration at the time of baseline evaluation. All patients were followed up every year via telephone or face-to-face interviews. For patients with a disease duration of less than 5 years, 227 patients with a follow-up period of at least 2 years were enrolled to analyze the impact of camptocormia on disability. The patients with disability during the baseline assessment were excluded (Fig. 2).

All the methods were carried out in accordance with relevant guidelines and regulations.

### Statistical analysis

First, because some of the data did not meet the homogeneity of variance and normality assumption required by the standard t-test, the Mann-Whitney U test was used to compare the continuous variables between patients with and without camptocormia, and the chi-squared test or Fisher’s exact test was used to compare categorical variables between patients with and without camptocormia (false discovery rate (FDR), corrected for multiple comparisons). In multiple comparisons, the FDR is a method to control the type I error rate in null hypothesis testing [19]. Then, the variables with a *P* value < 0.05 between the two groups and factors that had been previously reported to be related to camptocormia were further included as independent variables in the binary regression analysis to explore the potential factors related to camptocormia. This included MSA subtype (MSA-*P* = 1, MSA-C = 0), age, age of onset, disease duration, score of UMSARS-I, UMSARS-II, UMSARS-IV, total UMSARS, FAB score, HDRS score, and LEDD. The presence or absence of camptocormia was used as the dependent variable. Thereafter, to explore the impact of camptocormia on the disability in patients with a disease duration of < 5 years, 227 patients with a follow-up period of at least 2 years were enrolled in the propensity score matching (PSM) analysis, which included 31 patients with camptocormia. One-to-three matching was performed based on the nearest-neighbor matching, and the caliper width was 0.5. A total of 92 patients (29 with camptocormia and 63 matched pairs) were included in the disability analysis. The impact of camptocormia on disability was assessed by the stratified Cox regression model, which stratified by matching pairs. The age, MSA subtype (MSA-*P* = 1, MSA-C = 0), disease duration, OH, and total score of UMSARS were considered as adjusted factors. Two-tailed *P*-values of < 0.05, were considered statistically significant. IBM SPSS software (version 26.0) was used for statistical analysis.

## Results

Demographic and clinical features of MSA patients with and without camptocormia are shown in Table 1. A total of 716 patients with MSA were enrolled, including 352 (49.2%) MSA-P and 364 (50.8%) MSA-C. The mean age and disease duration were 60.18 ± 8.99, and 2.59 ± 1.69 years, respectively. There were 90 (12.6%) patients with camptocormia. Patients with camptocormia had a higher proportion of MSA-P subtype, older age, older age of onset, longer disease duration, higher proportion of symptom of onset with parkinsonism, lower score of FAB, higher score of HDRS, and higher LEDD than those without camptocormia (adjusted *P* < 0.05). After adjusting for age of onset and disease duration, Patients with camptocormia had a higher score of UMSARS-I, UMSARS-II, UMSARS-IV, and total UMSARS compared to those without camptocormia (adjusted P < 0.05). We found that the frequency of camptocormia was 8.9, 19.7, and 19.2% when the disease durations were ≤ 3, > 3 and < 5, and ≥ 5 years, respectively.

**Table 1:** Demographic and clinical features of the MSA patients with and without camptocormia

Variables	Total	With camptocormia	Without camptocormia	*P* value
Number	716	90 (12.6%)	626 (87.4%)	–
Diagnosis (MSA-P/MSA-C)	352/364	60/30	292/334	< 0.001*
Sex (male/female)	405/311	46/44	359/267	0.264
Age	60.18 ± 8.99	63.73 ± 7.91	59.67 ± 9.03	< 0.001*
Age of onset	57.54 ± 8.94	60.53 ± 7.81	57.11 ± 9.02	0.001*
Disease duration	2.59 ± 1.69	3.18 ± 1.67	2.50 ± 1.67	< 0.001*
Educational year	9.46 ± 3.92	9.21 ± 4.16	9.49 ± 3.89	0.522
Onset symptoms				
Autonomic symptom	247	28	219	0.005*
Cerebellar symptom	198	15	183	
Parkinsonism symptom	271	47	224	
UMSARS-I	14.30 ± 6.68	17.97 ± 7.43	13.77 ± 6.40	< 0.001*^#^
UMSARS-II	16.98 ± 6.95	21.89 ± 7.60	16.28 ± 6.56	< 0.001*^#^
UMSARS-IV	2.00 ± 0.96	2.68 ± 1.17	1.90 ± 0.88	< 0.001*^#^
Total UMSARS score	31.28 ± 12.80	39.86 ± 14.22	30.04 ± 12.11	< 0.001*^#^
OH (yes/no)	198/518	28/62	170/456	0.433
Total score of FAB	14.32 ± 2.96	13.52 ± 3.47	14.44 ± 2.87	0.006*
Total MOCA score	21.57 ± 5.06	20.71 ± 6.00	21.70 ± 4.91	0.084
HDRS score	11.47 ± 8.03	13.57 ± 9.09	11.16 ± 7.83	0.008*
HARS score	9.52 ± 7.02	10.11 ± 7.27	9.43 ± 6.98	0.392
LEDD (mg/day)	137.40 ± 243.00	209.58 ± 270.48	127.02 ± 237.22	0.001*

*MSA* multiple system atrophy, *MSA-P* multiple system atrophy with predominately parkinsonism, *MSA-C* multiple system atrophy with predominately cerebellar ataxia, *UMSARS* unified multiple system atrophy rating scale, *OH* orthostatic hypotension, *FAB* frontal lobe battery, *MoCA* Montreal cognitive assessment, *HDRS* Hamilton depression rating scale, *HARS* Hamilton anxiety rating scale, *LEDD* levodopa equivalent daily dose

*: significant difference after adjusting by false discovery rate

^#^: after adjusting for age of onset and disease duration

In the disease duration ≤3 years group, MSA-P patients were more common in the patients with camptocormia than that in patients without camptocormia (adjusted *P* < 0.05). Patients with camptocormia were older and had an older age of onset than patients without camptocormia (adjusted *P* < 0.05). The UMSARS-I, UMSARS-II, UMSARS-IV, and total UMSARS were higher in patients with camptocormia than in those without after adjusting for age of onset and disease duration (adjusted P < 0.05). Patients with camptocormia had lower FAB scores and higher HDRS scores than those without camptocormia (adjusted P < 0.05). (Table 2).

**Table 2:** Demographic and clinical features of the MSA patients with and without camptocormia according to different disease durations

Variables	Disease duration ≤3 years			Disease duration 3–5 years			Disease duration ≥5 years		
	With camptocormia	Without camptocormia	*P* value	With camptocormia	Without camptocormia	*P* value	With camptocormia	Without camptocormia	*P* value
Number	42 (8.9%)	428 (91.1%)	–	34 (19.7%)	139 (80.3%)	–	14 (19.2%)	59 (80.8%)	–
Diagnosis (MSA-P/MSA-C)	26/16	185/243	0.020*	23/11	76/63	0.171	11/3	31/28	0.077
Sex (male/female)	17/25	238/190	0.060	20/14	81/58	0.953	9/5	40/19	0.802
Age	63.23 ± 7.94	58.65 ± 8.50	0.001*	63.79 ± 8.48	62.50 ± 9.65	0.539	65.11 ± 6.49	60.40 ± 9.87	0.073
Age of onset	61.38 ± 7.87	56.99 ± 8.51	0.001*	60.02 ± 8.17	58.72 ± 9.65	0.595	59.19 ± 6.96	54.27 ± 10.31	0.062
Disease duration	1.83 ± 0.74	1.59 ± 0.73	0.040	3.68 ± 0.58	3.76 ± 0.62	0.614	6.02 ± 1.13	6.14 ± 1.36	0.861
Educational year	9.38 ± 4.13	9.49 ± 3.90	0.809	9.03 ± 4.28	9.36 ± 3.71	0.523	9.14 ± 4.24	9.86 ± 4.29	0.572
Onset symptoms									
Autonomic symptom	13	134	0.094	10	58	0.146	5	27	0.608
Cerebellar symptom	8	143		5	29		2	11	
Parkinsonism symptom	21	151		19	52		7	21	
UMSARS-I	16.64 ± 6.28	12.35 ± 5.97	< 0.001*^#^	17.94 ± 7.33	16.54 ± 5.88	0.340	22.00 ± 9.72	17.56 ± 6.93	0.157
UMSARS-II	20.79 ± 6.85	15.15 ± 6.33	< 0.001*^#^	21.53 ± 8.18	18.72 ± 5.94	0.142	26.07 ± 7.36	18.66 ± 7.49	0.003*
UMSARS-IV	2.52 ± 1.04	1.72 ± 0.74	< 0.001*^#^	2.68 ± 1.17	2.22 ± 1.01	0.036	3.14 ± 1.46	2.46 ± 1.07	0.093
Total UMSARS score	37.43 ± 12.10	27.50 ± 11.41	< 0.001 *^#^	39.47 ± 14.93	35.26 ± 10.85	0.207	48.07 ± 16.31	36.22 ± 13.74	0.027
OH (yes/no)	10/32	107/321	0.865	13/21	43/96	0.415	5/9	20/39	1.000
Total score of FAB	12.81 ± 3.80	14.49 ± 2.93	0.007*	14.18 ± 2.71	14.22 ± 2.65	0.962	14.07 ± 3.85	14.56 ± 2.91	0.843
Total MOCA score	20.05 ± 6.51	21.79 ± 4.95	0.197	21.88 ± 4.99	21.36 ± 4.74	0.568	19.86 ± 6.60	21.81 ± 5.03	0.500
HDRS score	13.14 ± 7.86	10.39 ± 7.68	0.025*	13.24 ± 9.63	12.10 ± 7.57	0.748	15.64 ± 11.41	14.54 ± 8.43	0.972
HARS score	9.71 ± 6.63	8.69 ± 6.71	0.256	9.32 ± 7.65	10.75 ± 7.15	0.163	13.21 ± 7.91	11.73 ± 7.69	0.457
LEDD (mg/day)	135.12 ± 240.37	97.52 ± 204.27	0.347	256.61 ± 263.95	185.07 ± 262.41	0.070	318 ± 324.44	204.24 ± 338.86	0.118

*MSA* multiple system atrophy, *MSA-P* multiple system atrophy with predominately parkinsonism, *MSA-C* multiple system atrophy with predominately cerebellar ataxia, *UMSARS* unified multiple system atrophy rating scale, *OH* orthostatic hypotension, *FAB* frontal lobe battery, *MoCA* Montreal cognitive assessment, *HDRS* Hamilton depression rating scale, *HARS* Hamilton anxiety rating scale, *LEDD* levodopa equivalent daily dose

*: significant difference after adjusting by false discovery rate

^#^: after adjusting for age of onset and disease duration

In the disease duration 3–5 years group, we found that there were no significant differences in the demographic and clinical features between the patients with and without camptocormia after adjusting by FDR. In the disease duration ≥5 years group, patients with camptocormia had higher UMSARS-II scores than patients without camptocormia (adjusted *P* < 0.05). (Table 2).

The factors associated with camptocormia in MSA patients with different disease durations are shown in Table 3. In the binary logistic regression model, older age of onset (OR 1.035, 95% CI 1.007–1.064, *P* = 0.013), MSA-P subtype (OR = 1.997, 95% CI 1.227–3.250 *P* = 0.005), and higher total UMSARS score (OR 1.056, 95% CI 1.038–1.075, *P* < 0.001) were related to camptocormia in the whole MSA. In addition, older age of onset (OR 1.047, 95% CI 1.002–1.094, *P* = 0.042), MSA-P subtype (OR 2.043, 95% CI 1.021–4.088, *P* = 0.043), higher total UMSARS score (OR 1.063, 95% CI 1.034–1.093, P < 0.001), and lower FAB score (OR 0.899, 95% CI 0.810–0.998, *P* = 0.046) were related to camptocormia in MSA patients in the ≤3 years disease group. A higher UMSARS-IV score was related to camptocormia in the disease duration 3–5 years group (OR 1.494, 95% CI 1.052–2.122, *P* = 0.025). A higher total UMSARS score was related to camptocormia in the disease duration ≥5 years group (OR 1.076, 95% CI 1.022–1.132, *P* = 0.005).

**Table 3:** Factors associated with camptocormia in MSA patients with different disease durations

Variables	Total MSA			Disease duration ≤3 years			Disease duration 3–5 years			Disease duration ≥5 years		
	OR	95% CI	*P* value	OR	95% CI	*P* value	OR	95% CI	*P* value	OR	95% CI	*P* value
Age of onset	1.035	1.007–1.064	0.013*	1.047	1.002–1.094	0.042*	–	–	–	–	–	–
Diagnosis (MSA-P = 1)	1.997	1.227–3.250	0.005*	2.043	1.021–4.088	0.043*	–	–	–	–	–	–
UMSARS total score	1.056	1.038–1.075	< 0.001*	1.063	1.034–1.093	< 0.001*	–	–	–	1.076	1.022–1.132	0.005*
FAB total score	-	-	-	0.899	0.810–0.998	0.046*	–	–	–	–	–	–
UMSARS-IV	-	-	-	–	–	–	1.494	1.052–2.122	0.025*	–	–	–

*MSA* multiple system atrophy, *MSA-P* multiple system atrophy with predominately parkinsonism, *MSA-C* multiple system atrophy with predominately cerebellar ataxia, *UMSARS* unified multiple system atrophy rating scale, *FAB* frontal lobe battery

*: significant difference

Furthermore, after PSM analysis, 92 patients included in the study which comprised of 29 patients with camptocormia and 63 matched pairs. The demographics and clinical features at baseline between patients with and without camptocormia during the disease duration of < 5 years were comparable, as shown in Table 4. The impact of camptocormia on disability in MSA patients with a disease duration of < 5 years is shown in Table 5. After adjusting for age, MSA subtype, disease duration, OH, and total score of UMSARS, camptocormia was found to have no significant impact on disability (HR 0.687; 95% CI, 0.252–1.874; *P* = 0.463).

**Table 4:** Comparison of demographics and clinical features at baseline between patients with camptocormia and matched pairs groups when the disease duration < 5 years

Variables	Total	With camptocormia	Matched pairs	*P* value
Number	92	29	63	–
Diagnosis (MSA-P/MSA-C)	50/42	17/12	33/30	0.577
Sex (male/female)	46/46	12/17	34/29	0.262
Age	62.30 ± 8.51	61.64 ± 7.53	62.61 ± 8.96	0.542
Age of onset	59.39 ± 8.47	58.63 ± 7.54	59.74 ± 8.91	0.427
Disease duration	2.91 ± 1.08	3.01 ± 1.01	2.87 ± 1.11	0.548
Educational year	10.13 ± 3.92	10.69 ± 4.31	9.87 ± 3.74	0.329
Onset symptoms				
Autonomic symptom	36	12	24	
Cerebellar symptom	23	5	18	0.488
Parkinsonism symptom	33	12	21	
UMSARS-I	17.99 ± 6.70	18.72 ± 7.06	17.65 ± 6.56	0.495
UMSARS-II	20.59 ± 7.51	21.38 ± 8.65	20.22 ± 6.96	0.749
UMSARS-IV	2.58 ± 0.97	2.72 ± 1.16	2.51 ± 0.88	0.375
Total UMSARS score	41.15 ± 13.93	42.83 ± 15.94	40.38 ± 12.97	0.699
OH (yes/no)	28/64	9/20	19/44	0.932
Total score of FAB	14.27 ± 2.87	14.59 ± 2.90	14.13 ± 2.87	0.462
Total MOCA score	21.59 ± 5.18	22.10 ± 5.48	21.35 ± 5.07	0.365
HDRS score	12.65 ± 8.40	13.24 ± 8.58	12.38 ± 8.37	0.680
HARS score	10.41 ± 7.12	10.59 ± 6.75	10.33 ± 7.34	0.893
Disability (yes/no)	45/47	12/17	33/30	0.327

*MSA-P* multiple system atrophy with predominately parkinsonism, *MSA-C* multiple system atrophy with predominately cerebellar ataxia, *UMSARS* unified multiple system atrophy rating scale, *OH* orthostatic hypotension, *FAB* frontal lobe battery, *MoCA* Montreal cognitive assessment, *HDRS* Hamilton depression rating scale, *HARS* Hamilton anxiety rating scale

**Table 5:** The impact of camptocormia on disability in MSA patients when the disease duration < 5 years

Variables	HR	95%CI	P value^#^
camptocormia	0.687	0.252–1.874	0.463

*MSA* multiple system atrophy

^#^: after adjusting for age, MSA subtype, disease duration, OH, and total score of UMSARS

## Discussion

To the best of our knowledge, this is the first study at this scale that focused on camptocormia in MSA patients with different disease durations. The frequency of camptocormia increased with prolonged disease duration, which was 8.9, 19.7, and 19.2% when the disease duration was ≤3, 3–5, and ≥ 5 years, respectively. The older age of onset, the subtype of MSA-P, and lower FAB score were associated with camptocormia in the early stage of the disease, in addition to disease severity, which was associated with camptocormia at different disease stages.

In the current study, camptocormia occurred in 12.6% of all patients with MSA, which was similar to the 15.6% reported in our previous study [10]. However, the frequency of camptocormia in MSA was lower than 26.3% (5/19) [3] and 32.1% (24/74) [4], and higher than 3.1% (5/160) [7]. The limitation of small sample size and heterogeneity of enrolled MSA patients of previous studies also supported that our finding can represent the real frequency of camptocormia in patients with MSA.

Furthermore, we found that the frequency of camptocormia increased with prolonged disease duration. Patients with camptocormia in our study had higher UMSARS scores among different disease durations, and disease severity was related to camptocormia among different disease durations using a binary logistic regression model. Altogether, these observations may indicate that the occurrence of camptocormia is a marker for disease progression. The pathogenesis of camptocormia is complex and not clearly understood. The possible pathogenesis of camptocormia in PD has been proposed as a part of disease progression [20], which might be involved in basal ganglia pathology [21]. A significant improvement in camptocormia after subthalamic nucleus stimulation has been reported [22], further supporting basal ganglia pathology as a cause of camptocormia. We also observed that patients with camptocormia were more common amongst individuals with MSA-P. Meanwhile, the MSA-P subtype was proven to be related to camptocormia in the binary logistic regression model, especially in the early stage of the disease. Glial cytoplasmic inclusions (GCIs) are more associated with striatonigral degeneration in MSA-P [23]. Our current findings suggest that basal ganglia pathology plays an important role in the development of camptocormia in MSA patients. The pathogenesis of camptocormia can also be explained by paraspinal myopathy or dystonia [20].

Additionally, we found that older age of onset was related to camptocormia in patients at the early stage of MSA. A Japanese study reported that camptocormia was associated with older age of onset in patients with PD [24], which partially supported our findings. Interestingly, we also found that a low FAB score was associated with camptocormia in the early stage of MSA in a binary logistic regression model, which has never been reported in patients with MSA. The association between cognitive impairment, such as orientation impairment and camptocormia, has been observed in PD patients [25, 26]. The current study found that frontal lobe dysfunction was associated with camptocormia rather than global cognitive impairment, which might be due to the frontal executive function being the most frequently affected cognition domain in MSA patients [27]. The mechanism of frontal lobe function impairment in camptocormia is unclear, and further studies are needed to clarify the same. In the current study, we did not find differences in symptoms of depression and anxiety between patients with and without camptocormia. We found that the above non-motor symptoms were not associated to camptocormia in MSA.

This is the first study to explore the factors related to camptocormia at different disease duration. We found that older age of onset, MSA-P subtype, and lower FAB score were associated with camptocormia only at an early stage of the disease, while greater disease severity was associated with camptocormia in all different disease stages. The severity of the disease gradually worsened with prolonged disease duration. We proposed that the effect of the severity of the disease on camptocormia is greater than that of cognition, age of onset and type of diagnosis at the middle or later stage of the disease. Additionally, there is a substantial overlap of cerebellar and parkinsonian features of MSA, indicating mixed neuropathological findings of GCIs distribution and neuronal loss in the striatonigral and olivopontocerebellar pathways [28]. Approximately 50% of MSA-P patients exhibited additional cerebellar signs, and over 50% of MSA-C patients developed parkinsonism with prolonged disease duration [29, 30]. Thus, the effect of type of diagnosis on camptocormia was not significant at the middle or later stage of the disease.

There is a dearth of literature that is focused on the relationship between camptocormia and disability in patients with MSA. In the current study, we found that camptocormia might be a marker of disease progression, but we failed to demonstrate the impact of camptocormia on disability in the Cox regression model. This could be due to the small sample size involved in the Cox analysis. Thus, in the future, large sample studies are needed to clarify the impact of camptocormia on prognosis.

There are some limitations to the present study. First, the follow-up time may not be sufficiently long. Further studies with longer follow-up times would help clarify the relationship between camptocormia and disability in patients with MSA. Second, the patients were registered from a single center, and further multicenter cooperation can reduce the selection bias. Third, electromyography (EMG) and MRI were not performed in patients with camptocormia in the current study. We will work on such issue in our future study to explore the potential peripheral mechanism of camptocormia in MSA through EMG and MRI.

## Conclusion

The present study involving a large cohort of patients with MSA demonstrates that camptocormia is common in patients with MSA. The older age of onset, MSA-P subtype, and lower FAB score are factors associated with camptocormia in the early stage of the disease, and disease severity is associated with camptocormia at different disease stages.

## Data Availability

All data generated or analyzed during this study are available from the corresponding author by reasonable request.

## Abbreviations

MSA: Multiple system atrophy
PD: Parkinson’s disease
SCA: Spinocerebellar ataxia
UMSARS: Unified Rating MSA Scale
OH: Orthostatic hypotension
FAB: Frontal assessment battery
MoCA: Montreal Cognitive Assessment
HDRS: Hamilton Depression Rating Scale
HARS: Hamilton Anxiety Rating Scale
FDR: False discovery rate
PSM: Propensity score matching
GCIs: Glial cytoplasmic inclusions
